# Generalized Dystonia Due to a Pathogenic *THAP1* Variant Showing Sustained Response to Globus Pallidus Deep Brain Stimulation

**DOI:** 10.5334/tohm.774

**Published:** 2023-08-22

**Authors:** Shakya Bhattacharjee, Monty A. Silverdale, Michael Bonello, Julian Evans, Christopher Kobylecki

**Affiliations:** 1 Department of Neurology, Manchester Centre for Clinical Neurosciences, Northern Care Alliance NHS Foundation Trust, Salford, UK; 2 Division of Neuroscience and Experimental Psychology, Manchester Academic Health Science Centre, University of Manchester, Manchester, UK; 3 Department of Neurology, The Walton Centre NHS Foundation Trust, Liverpool, UK; 4 Department of Neurosurgery, Manchester Centre for Clinical Neurosciences, Northern Care Alliance NHS Foundation Trust, Salford, UK

**Keywords:** dystonia, THAP1, novel, mutation, deep brain stimulation

## Abstract

A 21-year-old woman of south Asian origin presented with cervical dystonia which had progressed over the previous three years. Her symptoms started as writer’s cramp since the age of seven years. She did not respond to medications and needed botulinum toxin injection for generalised dystonia. Subsequent whole genome sequencing revealed a likely pathogenic c.98G>A p.(Cys33Tyr) heterozygous variant in the *THAP1* gene. She underwent bilateral posteroventral globus pallidus interna (GPi) deep brain stimulation (Medtronic Activa PC) implantation at the age of thirty-one years. She responded well to the deep brain stimulation even after more than 8 years post-surgery though she needs botulinum toxin injection for her cervical dystonia.

Genetic variants in *THAP1* are the second most common cause of isolated monogenic dystonia after *TOR1A* [1]. However, the reported response to deep brain stimulation (DBS) is less clearly defined, making it harder to counsel patients referred for surgery [2]. We report a patient with generalized dystonia typical of DYT-*THAP1* with a previously reported variant now classed as pathogenic, with a very good response to globus pallidus interna (GPi) DBS after almost nine years of surgery demonstrated by video assessment.

## Case report

A 21-year-old woman of south Asian origin presented with cervical dystonia which had progressed over the previous three years. Her symptoms started as writer’s cramp since the age of seven years and had progressed with overflow movements. She was treated with tetrabenazine up to 25 mg three times daily and trihexyphenidyl up to 2 mg TDS but they were ineffective and caused side effects. Botulinum toxin (BoNT) type A injections for cervical and upper limb dystonia were initially effective, but the duration of benefit progressively declined despite abobotulinum toxin A (Dysport, Ipsen pharmaceutical limited) dose of 250 units in the right sternomastoid and left splenius capitis each, 150 units in the left levator scapulae, and 100 units in the left trapezius muscle. She developed dystonic posturing of the left lower limb and laryngeal involvement in her mid-20s and was referred for deep brain stimulation (DBS) surgery at the age of 30.

She underwent bilateral posteroventral globus pallidus interna (GPi) DBS (Medtronic Activa PC) implantation at the age of 31 (settings in Table 1). The location of the leads was within target ranges of both GPi and the locations of leads were confirmed by the perioperative imaging. (Supplementary figure) Unipolar stimulation of the DBS created side effects so bipolar stimulation was used. Over the years the voltage was gradually increased as shown in the tables with no change in the pulse width or frequency as that provided the optimal result for the patient. The pre-surgery Toronto Western Spasmodic Torticollis Rating Scale (TWSTRS) motor score was 26 (Supplementary video 1) and Global Dystonia Severity Rating Scale (GDS) score was 48. The TWSRTS scale score reduced to 11 and after 6 months of operation and to 16 after 2 years post-DBS. (supplementary table) The BoNT injections were continued to the neck and right upper limb. She reported significant benefit for both her cervical dystonia and upper limb dystonia, also evidenced by marked worsening when DBS was temporarily switched off for EMG-guided BoNT injections to the neck and upper limb muscles (supplementary videos 2 and 3). The sensory trick was no longer effective. At 8.5 years of follow up there is severe dystonic posturing in neck, trunk, and upper limbs in the DBS “OFF” state which improves once DBS is switched back on (supplementary videos 2 & 3). There had been a clear progression of the disease since the DBS surgery 8.5 years back though she feels functionally more independent (Supplementary table).

**Table 1:** Initial and most recent deep brain stimulation parameters.


PARAMETER	INITIAL SETTINGS		MOST RECENT SETTINGS	

	LEFT	RIGHT	LEFT	RIGHT

Contacts	1+ 2 –	10+ 11 –	1+ 2 –	10+ 11 –

Amplitude	2.5 V	2.5 V	3.1 V	2.9 V

Pulse width	60 µs	60 µs	60 µs	60 µs

Frequency	130 Hz	130 Hz	130 Hz	130 Hz


**Video 1:** Taken prior to DBS implantation at age 31. The video shows a marked left torticollis and right laterocollis, with elevation of the right shoulder and evidence of a sensory trick. There is dystonic posturing of the right upper limb and evidence of task-specific dystonia on writing.

**Video 2:** In DBS ‘ON’ stage at 40 (8.5 years post DBS implantation). There is moderate left torticollis with restricted range of movement to right, and anterior sagittal shift.

**Video 3:** In DBS “OFF” state. Severe left torticollis with inability to turn the head past the midline to the right, with additional severe antecaput and truncal involvement. Significant tremulous dystonic posturing of both upper limbs is evident.

Subsequent whole genome sequencing revealed a c.98G>A p.(Cys33Tyr) heterozygous variant in the *THAP1* gene. This variant is classed as likely pathogenic: it is absent from the controls of GnomAD database, the clinical presentation is consistent, and the gene product is predicted likely deleterious. Danielsson A et al, previously reported one patient with the same *THAP1* variant, classed as a variant of uncertain significance at that time [7].

## Discussion

DYT-*THAP1* was reported as a cause of monogenic isolated dystonia syndrome with a broad spectrum of genetic variants [1]. Pathogenic variants can affect all three exons of the gene. The mutations can affect THAP1 function, mainly its DNA-binding capacity and nuclear translocation.

The long-term motor response after pallidal DBS in DYT-*THAP1* patients had been unsatisfactory or variable, and some patients may even deteriorate after an initial good response [3,4,5,6]. The cause for the relatively lesser response to DBS might be due to the prominent bulbar involvement or the marked genetic heterogeneity of the *THAP1* mutation [1], compared to DYT-*TOR1A* in which most patients harbour the same genetic variant. More recent reports suggest a more favourable response to GPi DBS in 14 patients with DYT-*THAP1*, in whom 11 were classed as responders, with 58% reduction in Burke-Fahn-Marsden motor score (BFM-M) after a median follow-up of 4 years and 10 months [7]. The patient reported in that paper with the same *THAP1* variant as we report here showed 58% reduction in BFM-M score after only one year of follow-up [7]. Krause and colleagues showed 21–67% clinical improvement for up to 11 years of GPi DBS in two of three male patients with early onset generalized or segmental DYT-*THAP1* dystonia [3].

Other recent reports expand the spectrum of DBS response in DYT-*THAP1*. One patient from India with a novel *THAP1* frameshift deletion mutation (c.208-209delAA; p.K70VfsX15) also showed a positive response to GPi DBS even after ten years, with particular response of lower limb and truncal dystonia [8]. A recent report of a patient with a novel base pair deletion mutation in exon 3 of the *THAP1* gene showed a good response to GPi DBS; however, this was during only two months of follow up, unlike our patient who had a sustained DBS response even after over eight years post-implantation [9].

We report a *THAP1* variant previously classed as a variant of uncertain significance, now classified as pathogenic, with a prolonged and sustained response to GPi DBS. Given the marked clinical and genetic heterogeneity of DYT-*THAP1* and variability reported in DBS response, such reports add to the ability of clinicians to counsel prospective candidates for DBS in monogenic dystonia.

## Additional Files

The additional files for this article can be found as follows:

10.5334/tohm.774.s1 Supplementary Table. Supplementary table: Cervical and generalised dystonia severity scales calculation before and after deep brain stimulation (DBS) surgery.

10.5334/tohm.774.s2 Supplementary figure(s). Supplementary figure: preoperative planning of leads in the right (2A) and left globus pallidus interna (2B) and the post operative imaging of the placements of the leads in the right (2C) and left globus pallidus interna(2D). The right globus pallidus internal lead is within 2 mm of preoperative planning site and the left lead was exactly at the planned location in the left globus pallidus.
