# Supplementary material for: Generalized Dystonia Due to a Pathogenic THAP1 Variant Showing Sustained Response to Globus Pallidus Deep Brain Stimulation
Source: Tremor Other Hyperkinet Mov (N Y). 2023 Aug 22;13:23. doi: 10.5334/tohm.774 (PMC10453957; doi:10.5334/tohm.774)
Supplement: Supplementary Table. — Supplementary table: Cervical and generalised dystonia severity scales calculation before and after deep brain stimulation (DBS) surgery. [file tohm-13-1-774-s1.pdf]

Supplementary table: Cervical and generalised dystonia severity scales calculation before and after

Scale	Score during first consultation	6 months after DBS(on phase) score	24 months after DBS(on phase) score	DBS off (after 8.5 years of surgery) score	DBS on (after 8.5 years of surgery) score	Improvement between off and on state after 8.5 years (%)
TWSRTRS	26	11	16	27	14	48.1
GDSRS	48	Not available	Not available	70	34	51.4

deep brain stimulation (DBS) surgery

TWSTRS- Toronto Western Spasmodic Torticollis Rating Scale

GDS-Global dystonia severity rating scale

DBS-Deep brain stimulation
