# Supplementary figures and images for: Generalized Dystonia Due to a Pathogenic THAP1 Variant Showing Sustained Response to Globus Pallidus Deep Brain Stimulation
Source: Tremor Other Hyperkinet Mov (N Y). 2023 Aug 22;13:23. doi: 10.5334/tohm.774 (PMC10453957; doi:10.5334/tohm.774)

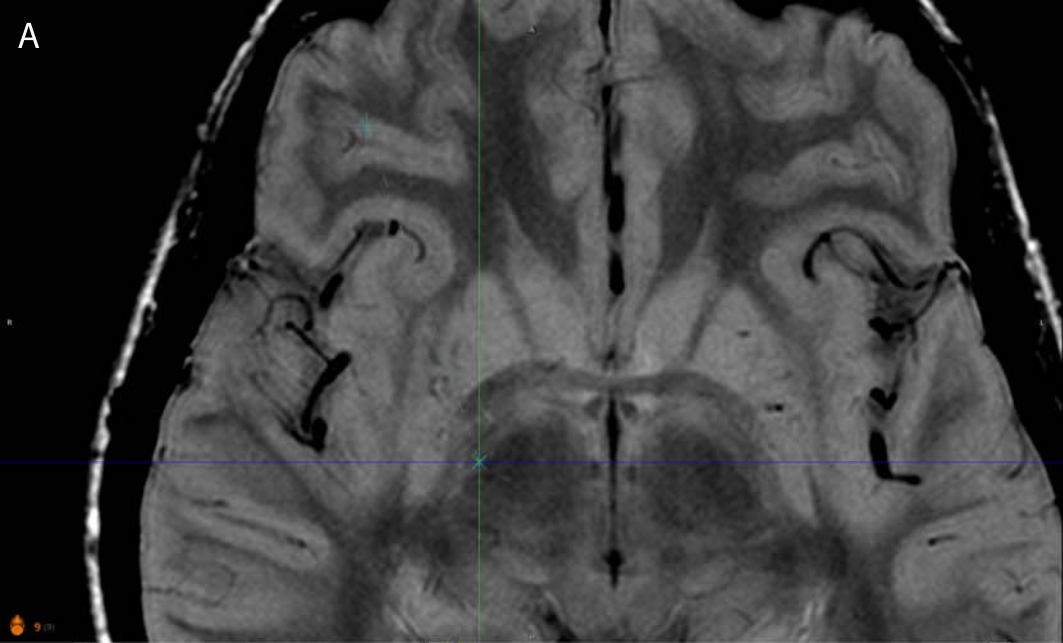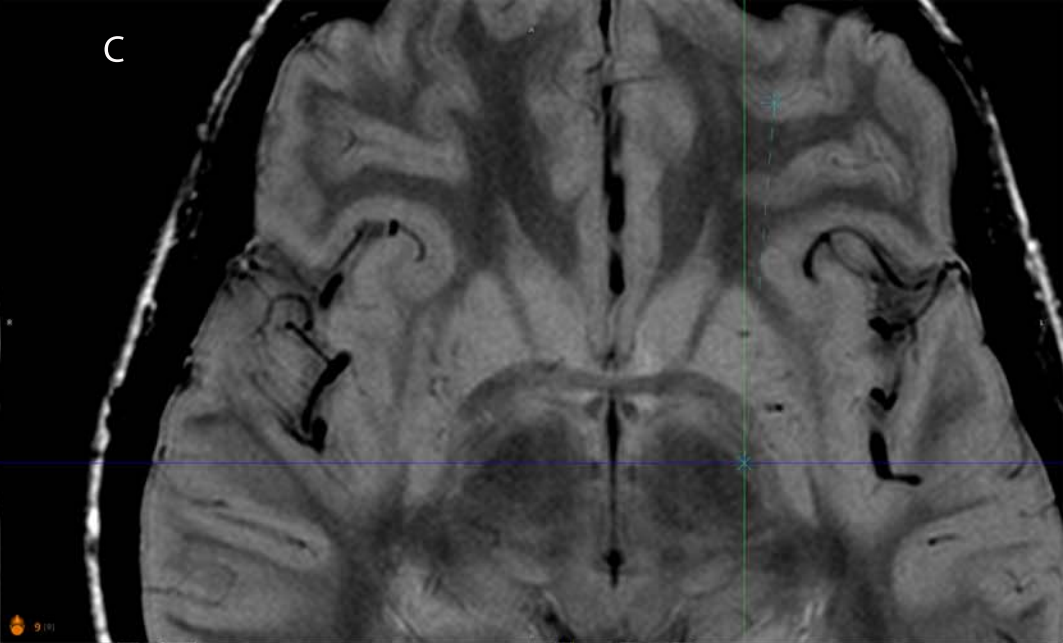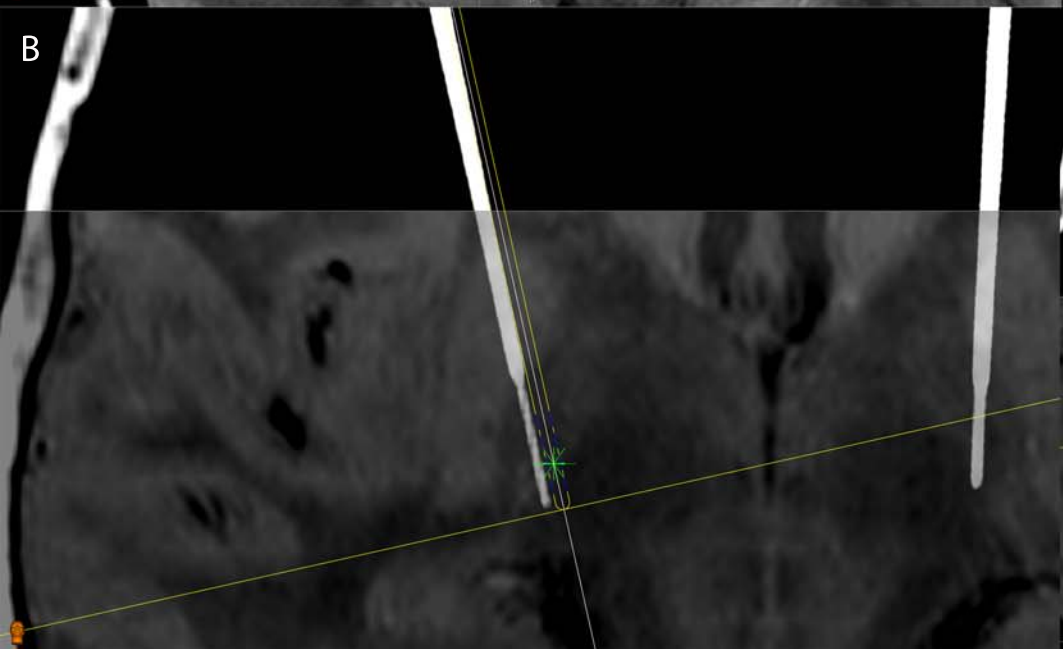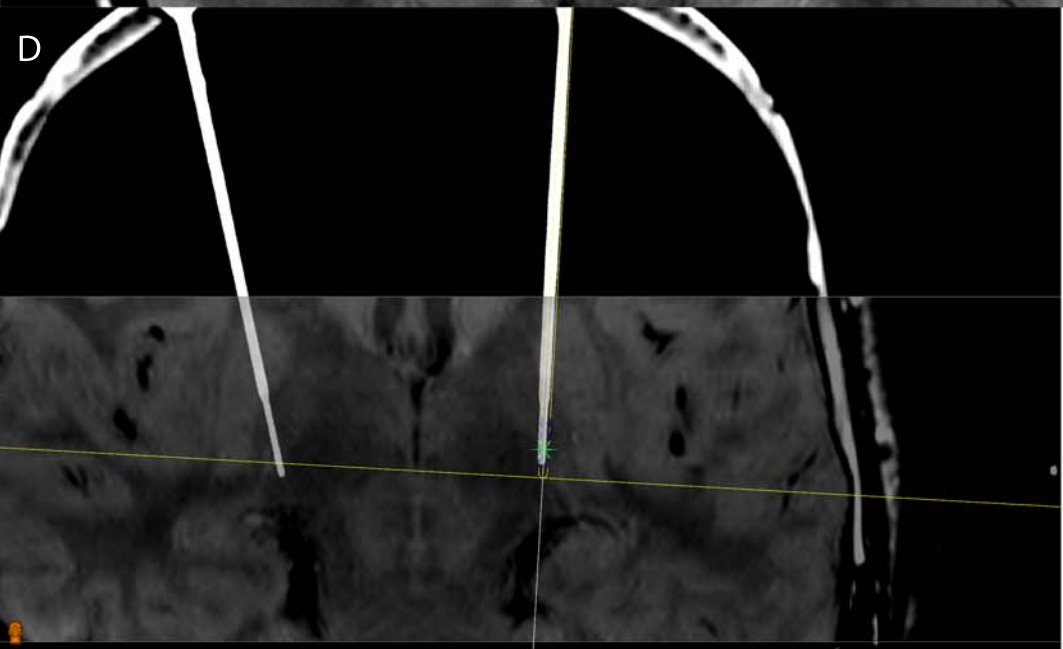

Supplement: Supplementary figure(s). — Supplementary figure: preoperative planning of leads in the right (2A) and left globus pallidus interna (2B) and the post operative imaging of the placements of the leads in the right (2C) and left globus pallidus interna(2D). The right globus pallidus internal lead is within 2 mm of preoperative planning site and the left lead was exactly at the planned location in the left globus pallidus. [file tohm-13-1-774-s2.pdf]
